# Reference Gene Selection in the Desert Plant *Eremosparton songoricum*

**DOI:** 10.3390/ijms13066944

**Published:** 2012-06-07

**Authors:** Xiao-Shuang Li, Hong-Lan Yang, Dao-Yuan Zhang, Yuan-Ming Zhang, Andrew J. Wood

**Affiliations:** 1 Key Laboratory of Biogeography and Bioresource in Arid Land, Xinjiang Institute of Ecology and Geography, Chinese Academy of Sciences, Urumqi 830011, China; E-Mails: xiaoshuangliok@sina.com (X.-S.L.); yhlan-163@163.com (H.-L.Y.); zhangym@ms.xjb.ac.cn (Y.-M.Z.); 2 Graduate University of Chinese Academy of Sciences, Beijing 100049, China; 3 Department of Plant Biology, Southern Illinois University, Carbondale, IL 62901, USA; E-Mail: wood@plant.siu.edu

**Keywords:** *Eremosparton songoricum*, quantitative real-time PCR, reference genes, geNorm, validation

## Abstract

*Eremosparton songoricum* (Litv.) Vass. (*E. songoricum*) is a rare and extremely drought-tolerant desert plant that holds promise as a model organism for the identification of genes associated with water deficit stress. Here, we cloned and evaluated the expression of eight candidate reference genes using quantitative real-time reverse transcriptase polymerase chain reactions. The expression of these candidate reference genes was analyzed in a diverse set of 20 samples including various *E. songoricum* plant tissues exposed to multiple environmental stresses. GeNorm analysis indicated that expression stability varied between the reference genes in the different experimental conditions, but the two most stable reference genes were sufficient for normalization in most conditions. *EsEF* and *Esα-TUB* were sufficient for various stress conditions, *EsEF* and *EsACT* were suitable for samples of differing germination stages, and *EsGAPDH*and *EsUBQ* were most stable across multiple adult tissue samples. The *Es18S* gene was unsuitable as a reference gene in our analysis. In addition, the expression level of the drought-stress related transcription factor *EsDREB2* verified the utility of *E. songoricum* reference genes and indicated that no single gene was adequate for normalization on its own. This is the first systematic report on the selection of reference genes in *E. songoricum*, and these data will facilitate future work on gene expression in this species.

## 1. Introduction

The drought-tolerant plant *Eremosparton songoricum* (Litv.) Vass. is a leafless perennial clone-forming semi-shrub [1]. *E. songoricum* is a rare endemic plant of central Asia and is the only species of the genus in China [2], where it is found only in the Gurbantunggut desert (Xinjiang, China [3]). As a rare and extremely drought-tolerant Leguminosae shrub, *E. songoricum* is being developed as a model organism for investigating the morphological, biochemical, physiological and molecular adaptations and responses to water deficit. The ability to identify and compare the expression profiles of key genes involved in these mechanisms would provide a powerful tool for evaluating the molecular changes in plants adapted to the desert environment. The *DREB* (dehydration-responsive element binding protein) gene plays a crucial role in plant stress response and signal transduction [4,5]. The *DREB2* gene family is particularly responsive to dehydration stress [5], with many plant species accumulating elevated amounts of its transcript in response to drought and water deficit stress [6–9].

Quantitative real-time reverse transcriptase polymerase chain reaction (qRT-PCR) technology has been widely used in gene expression analysis due to its quantitative accuracy, high sensitivity and high-throughput capabilities [10–13]. The accuracy of qRT-PCR can be influenced by several factors: the quality and quantity of the mRNA template, variations in reaction efficiency and differences between cells or tissues [14,15]. The selection of stable reference genes is a prerequisite for performing qRT-PCR. Recent reports demonstrate that qRT-PCR data must be normalized with one or more suitable and stable internal reference genes [16]. In the plants *Cichorium intybus* and *Brachiaria brizantha*, it has been suggested that at least two reference genes be combined to normalize the results of qRT-PCR [17,18]. Moreover, reference genes may show a different stability pattern even within the same plant, and the results of qRT-PCR cannot be extrapolated to other experimental conditions [19–21]. Consequently, it is recommended that suitable reference genes should be established for each species and tested to ensure their stability under the desired experimental conditions [22].

Many qRT-PCR expression studies have focused on humans and other animal model organisms [23–25]. In plants, stable reference genes have been identified for grasses [18,26–28], fruits [19,20], vegetables [29–36], and commercial agricultural crops [11,22,37–43]. However, few studies have been conducted using legumes, and almost all of the reports published have utilized herbaceous plants [21,44–48] and have not included *E. songoricum*.

In this study, we aimed to clone and evaluate candidate reference genes for expression studies in the Leguminosae shrubby plant *E. songoricum*. Samples were collected from different *E. songoricum* tissues at different developmental stages and exposed to different stress treatments. To illustrate the utility of the characterized reference genes, we conducted detailed expression analysis of the transcription factor *EsDREB2* in *E. songoricum*.

## 2. Results

### 2.1. Sequence Analysis of the Candidate Reference Genes

A single, partial cDNA fragment was cloned for each of these reference genes with the exception of *β-TUB*, for which two partial cDNA fragments were cloned (supplemental material). The cDNA fragments of the eight common reference genes *EsGAPDH*, *EsUBQ*, *EsACT*, *EsEF*, *Esβ-TUB1*, *Esβ-TUB2*, *Esα-TUB*, and *Es18S* ranged from 428 bp to 633 bp. BLASTN revealed that these amplified fragments had 85%–99% identity with similar sequences from other plant species, and six out of eight of these references genes had the maximum identity with a legume. For example, the *EsGAPDH* gene sequence had 91% identity with the *Medicago truncatula GAPDH* mRNA over a span of 574 bp (GenBank NoXM_003601780). All cDNA sequences were deposited to the GenBank database under accession numbers JN866814 to JN866821.

### 2.2. Verification of Primer Specificity and Efficiency

**Figure 1:**
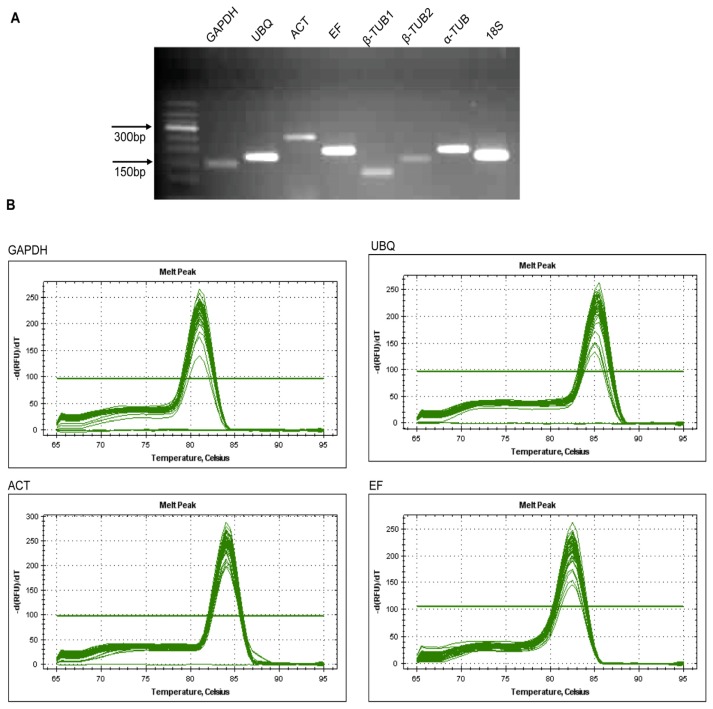

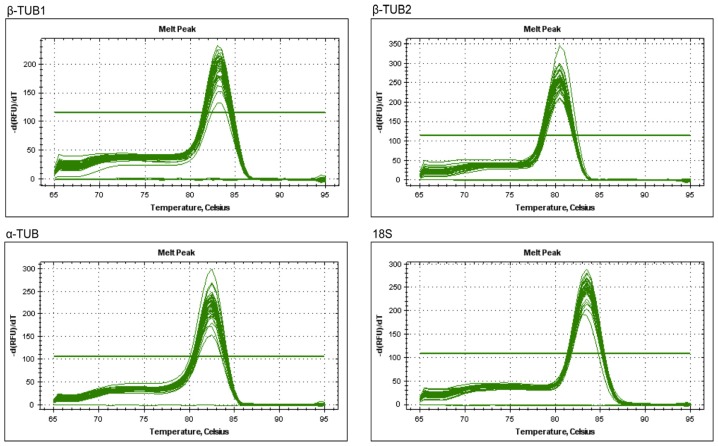
Specificity of qRT-PCR amplification. (**A**) Agarose gel (2.0%) showing the amplified products of the reference genes from qRT-PCR at the expected sizes. (**B**) Dissociation curves of eight reference genes showing single peaks for each including three technical replicates for each of the 20 cDNA pools of test samples.

The primer sequence and amplicon characteristics of eight candidate reference genes for qRT-PCR are described in Table 1. The primer efficiency for each primer pair was greater than 90%. The specificity of each primer pair was further confirmed by gel electrophoresis analysis of the qRT-PCR amplification products (each primer pair amplified only a single product of the predicted size), and melting curve analysis (a single peak was obtained) (Figure 1).

### 2.3. Expression Profiling of Reference Genes (qRT-PCR Assay)

**Figure 2:**
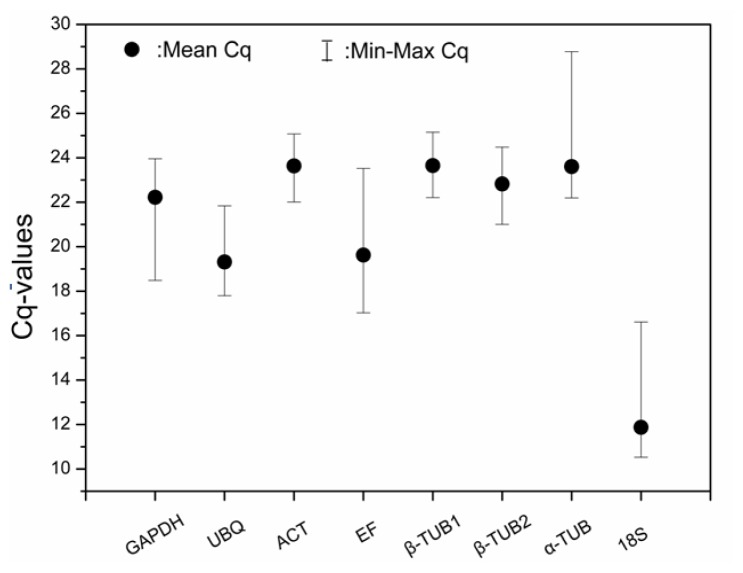
Expression levels of eight candidate reference genes in 20 different samples. The median value and the minimum and maximum *Cq* of the 20 samples were calculated.

Amplification of the *EsACT* gene in each of the 20 cDNA samples produced a single ACT-specific band with a predicted molecular size (approximately 300 bp) (data not shown), which confirmed that the RNA samples extracted from *E. songoricum* are appropriate for mRNA analysis. The expression of the eight reference genes from each RNA sample is shown in Figure 2. The median *Cq* value ranged from 10.53 to 28.78. *Esα-TUB* had the highest *Cq* value, which indicated relatively low expression. In contrast, *Es18S* had the highest expression level, and the threshold fluorescence was reached after approximately 12 cycles. Most *Cq* values were between 15 and 25 cycles, and the average *Cq* value of the samples (excluding *Es18S*) was 22 cycles.

### 2.4. GeNorm Analysis

**Figure 3:**
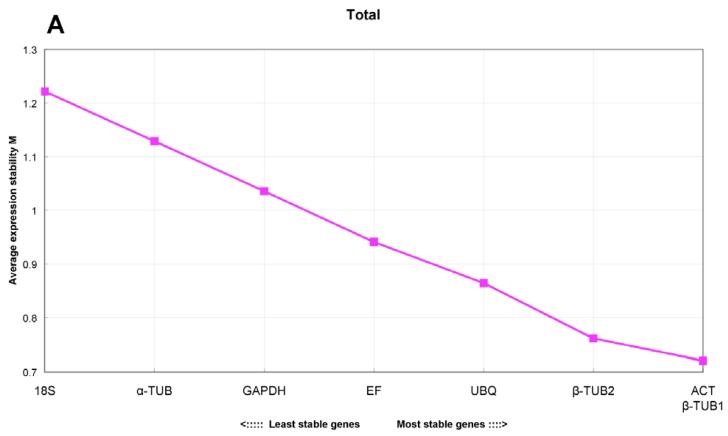

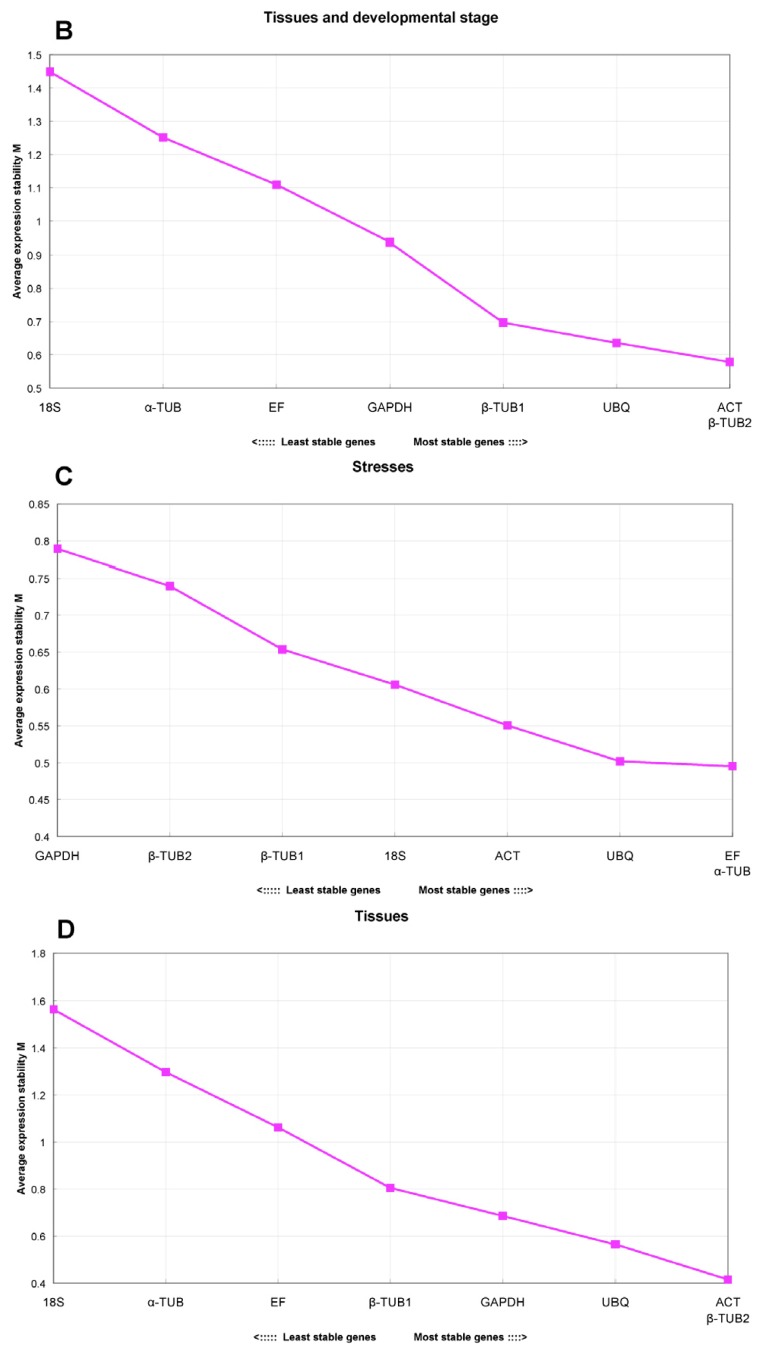

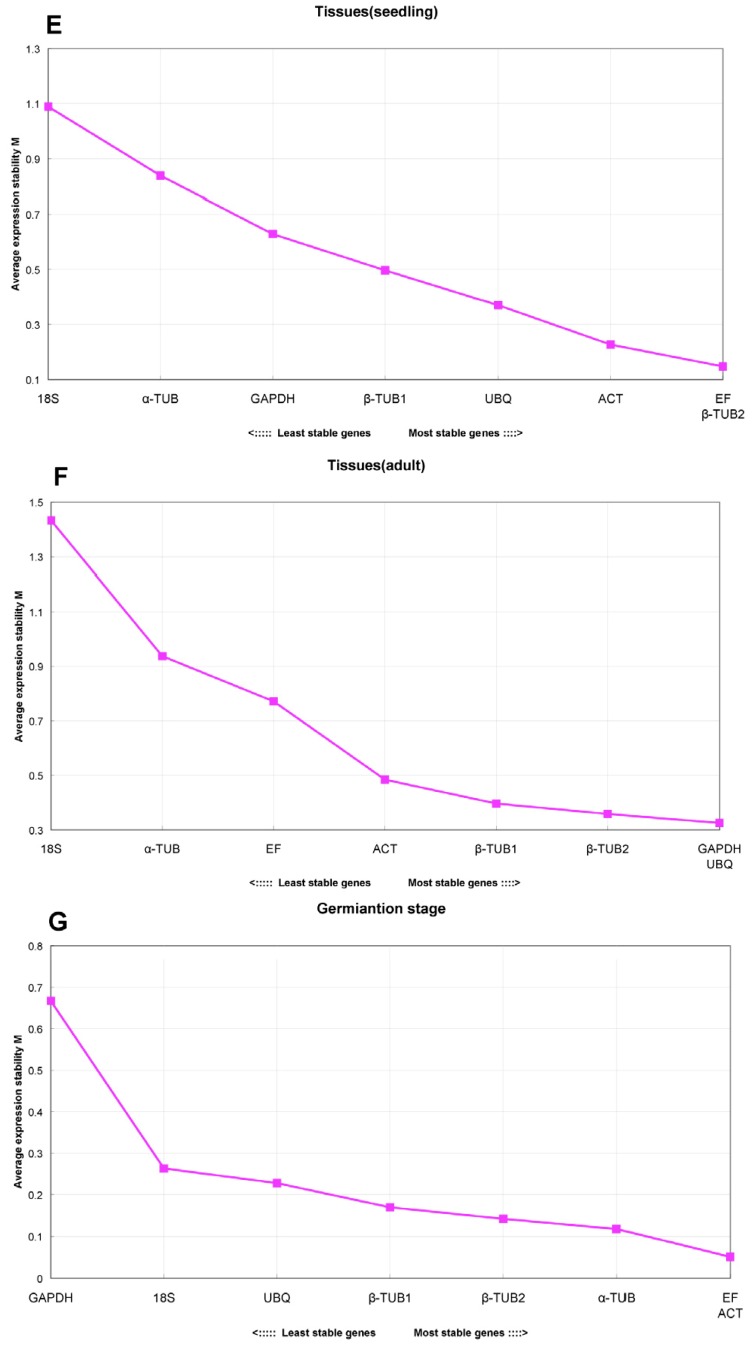
Average expression stability values (*M*) of the selected reference genes as calculated by geNorm. A lower value of average expression stability (*M*) indicated more stable gene expression; the cutoff for M was set as 1.5, as suggested by geNorm. (**A**) all tested samples; (**B**) tissues and developmental stage; (**C**) abiotic stresses, (**D**) all tissue samples (including adult and seedling samples); (**E**) seedling tissues (root, stem, leaf); (**F**) adult tissues (root, branch, flower); (**G**) germination stage.

GeNorm software was used to rank the eight reference genes based on the experimentally determined expression stability value *M*. Figure 3 shows the average expression values for the eight candidates under different conditions. According to the geNorm manual, the gene with the lowest *M* value is considered the most stable, while the highest *M* value indicates the least stable expression. All eight genes examined showed *M* values lower than the cut-off recommended by geNorm (*M* < 1.5). Across all the test samples, *EsACT* and *Esβ-TUB1* were considered to be the most stable genes, while *Es18S* and *Esα-TUB* were shown to be the least stable genes (Figure 3A). Across the different tissues and developmental stages, similar result was obtained that *EsACT* and *Esβ-TUB2* were the most stably expressed genes, while *Es18S* and *Esα-TUB* were the least stable (Figure 3B). For stress-treated samples, *EsEF* and *Esα-TUB* were the most stable reference genes, while *EsGAPDH* and *Esβ-TUB2* were the least stable (Figure 3C). When combining adult plant material from the field with seedling material grown under controlled conditions, *EsACT* and *Esβ-TUB2* genes demonstrated the best expression stability (Figure 3D).

The stability of the reference genes in a smaller experimental design was investigated. In root, stem and leaf samples of two-week-old seedlings grown under controlled conditions, the *EsEF* and *Esβ-TUB2* genes were the most stable (Figure 3E). However, for field-grown adult samples (adult roots, assimilating branches and flowers), *EsGAPDH* and *EsUBQ* were the most stable (Figure 3F). For the different germination stages, *EsEF* and *EsACT* were the most stable, with an M value less than 0.1 (Figure 3G). *Es18S* ranked poorly across all tested conditions, while *EsACT* always showed a stable expression cross all the samples.

**Figure 4:**
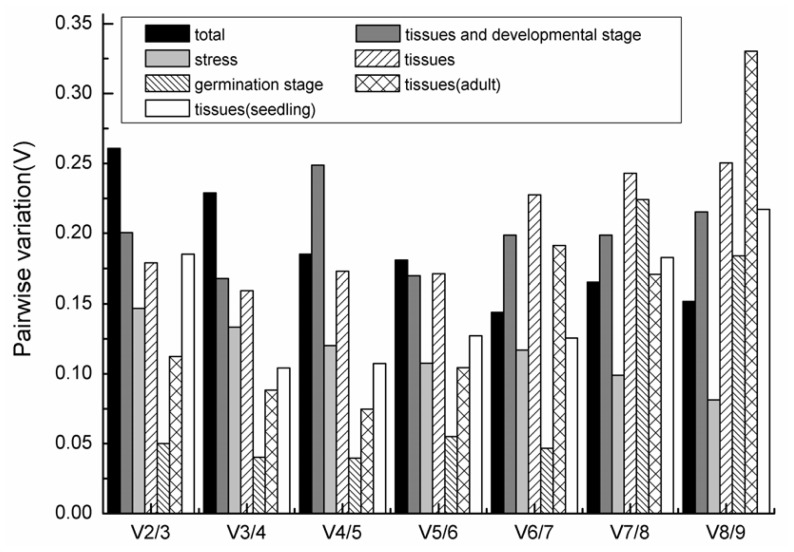
Pairwise variation (*V*) analyses of the candidate reference genes. The pairwise variation (*V_n_*_/_*_n+_*_1_) was analyzed for the normalization factors NF*_n_* and NF*_n_*_+1_ by the geNorm software to determine the optimal number of reference genes for accurate normalization. The cutoff value was proposed to be 0.15, below which the inclusion of an additional reference gene is not necessary.

To determine the optimal number of reference genes required for accurate normalization, we calculated the pairwise variation (*V_n_*_/_*_n+_*_1_) using geNorm. GeNorm uses 0.15 as the cut-off value, below which the inclusion of additional reference genes is not necessary. However, it has been suggested that this cut-off value is too strict [15]. In our analysis (Figure 4), for all the samples together, seven genes are required for proper normalization (*V*_7/8_ = 0.163). For samples from the different tissues and developmental stages, the *V*_3/4_ was 0.168, which is slightly greater than the recommended cutoff value (Figure 4). Under the stress conditions, the V_2/3_ value is below 0.15 (0.147), which indicated that only the most two stable reference genes *EsEF* and *Esα-TUB* are needed to reliably normalize the expression data (Figure 4). Considering all the tested tissue samples, *V*_4/5_ was 0.186, which is still higher than the cutoff, but when we analyzed smaller datasets, the *V* value improved in the seedling tissues (root, stem and leaf) and adult plant samples (branch, flower and root). The stability *M* value rank was slightly changed in seedling tissues and adult plant samples, but the pairwise variation (*V_n_*_/_*_n+_*_1_) was much lower compared to the whole tissue samples. Only the combinations of the *EsEF* and *Esβ-TUB2* (*V*_2/3_ = 0.087), *EsGAPDH* and *EsUBQ* (*V*_2/3_ = 0.113) genes were sufficient for reliable normalization of seedling tissues and adult plant tissues, respectively (Figure 4). Similar results were also found for the different stages of seed germination, only the most two stable genes (*EsEF* and *EsACT*) is enough for an accurate normalization result with the *V*_2/3_ value less than 0.05 (Figure 4). The most stable reference genes and the optimal gene combinations were not identical for the different experimental conditions. The results obtained by geNorm are summarized in Table 2.

### 2.5. *EsDREB2* Gene Expression

**Figure 5:**
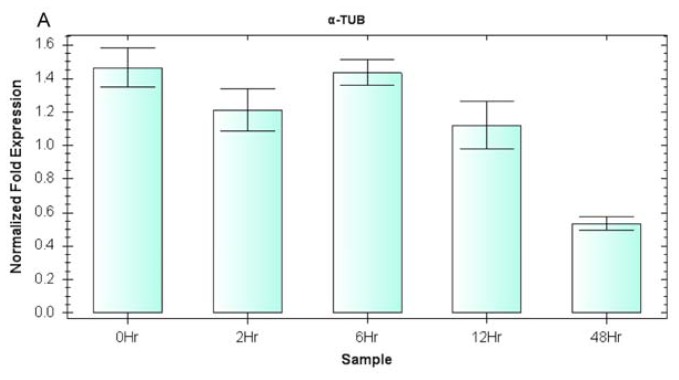

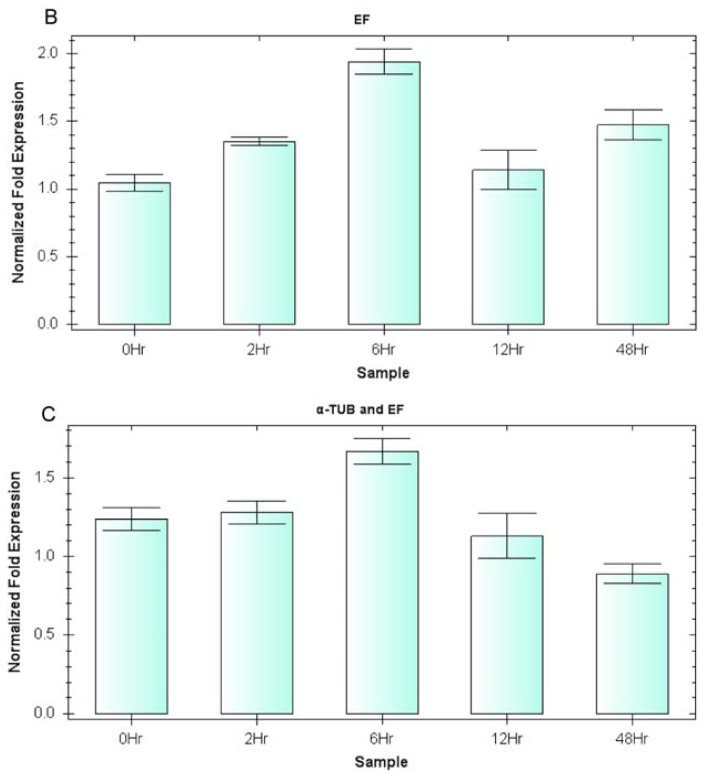
The expression of *EsDREB2* in response to drought stress. Relative gene expression was analyzed after plants were exposed to 0 h, 2 h, 6 h, 12 h and 48 h of drought stress conditions. Normalization was performed using α-tubulin (*α-TUB*) alone (**A**), *EF* alone (**B**) or the combination of *α-TUB* and *EF* (**C**).

The expression of the *EsDREB2* gene in response to drought stress was assessed using the one or two most stable reference gene(s) (*EF* and *α-TUB*) for normalization, which was validated by geNorm as described above and shown in Figures 3,4. The geometric average of the most stable reference genes in the stress treatment (*EF* and *α-TUB*) was used as an internal control. The expression pattern of *EsDREB2* did significantly change with the reference gene(s) used for normalization. With either *EsEF* used alone for normalization or *EsEF* and *Esα-TUB* combined together as reference genes, *EsDREB2* was found to be up-regulated by drought stress. The transcript gradually increased and peaked at 6 h and then began to decline at 12 h (Figure 5B,C). However, normalizing with *Esα-TUB* alone revealed that the expression level of *EsDREB2* was decreased by drought stress (though it slightly increased at 6 h) (Figure 5A).

## 3. Discussion

QRT-PCR is a powerful technology for gene expression studies and is predicated upon the utilization of suitable reference genes to ensure reliable and accurate data. In the present study, eight commonly used housekeeping genes were identified and evaluated as qRT-PCR candidate reference genes in the drought-tolerant plant *E. songoricum*. Our data indicated that the expression levels of these eight genes vary under different experimental conditions. The results also showed that a smaller experimental design improve the results. These results highlight the fact that reliable reference genes are highly specific to the experimental conditions, and thus it is important and necessary to carefully evaluate reference genes for every experimental design.

The stability of different members of the same gene family can vary greatly. Hu *et al*. [16] demonstrated that *ACT2/7* is less stable than *ACT11*, and the *TUA* gene is more stable than the *TUB* gene under many conditions. In the present study, we selected different members of the tubulin gene family including *Esα-TUB*, *Esβ-TUB1*, and *Esβ-TUB2* to compare their performance for normalization. The stability ranking of the tubulin gene family was as follows: *Esβ-TUB2* > *Esβ-TUB1* > *Esα-TUB*. This is particularly true for *β-TUB2*, which is always ranked among the three most stable genes, while for *Esα-TUB*, it is ranked poorly in most of the experimental conditions (Table 2).

Of the eight genes tested, the *Es18S* gene was the least stable across the experimental conditions (Figure 3). This gene is abundantly expressed compared with the average expression level of the other reference genes. There are a number of published reports that support this phenomenon (e.g., high expression of the *18S* gene) including data from cucumber and *Hevea brasiliensis* [30,49]. Interestingly, the widely used 18S rRNA gene ranked poorly for almost all experimental conditions [41,46–48], demonstrating that the *18S* gene is not suitable for normalization in legumes.

The actin gene is one of the most commonly used reference genes. Recent reports have increasingly argued that the actin gene might not be stably expressed under different conditions [20,28,35,50]. However, in the present study, the actin gene showed a high stability under all experiment conditions tested, demonstrating that actin is a suitable reference gene for qRT-PCR analysis in *E. songoricum.*

More recent studies have reported that novel reference genes such as HSP90 metalloprotease, ATP-binding cassette [ABC] transporter, and miRNAs outperform the traditional housekeeping genes in terms of expression stability [44,46,47]. A recent study in soybean [44] reported that CDPK-related protein kinase (*CDPK*) and F-box protein family (*F-box*) were stably expressed and suitable as reference genes, so these were tested in the present study as candidates in *E. songoricum.* The *F-box* gene amplicon was a smear using the reported primers, and the *CDPK* gene was found to have a low transcript level and unstable expression in the tested samples, so these two genes were not considered for further qRT-PCR analysis (data not shown). These two novel reference genes may be useful and important in future studies on *Eremosparton songoricum* gene expression analysis. The combination of these novel reference genes and classic traditional house-keeping genes may be a good strategy for qRT-PCR based gene expression studies.

Increasing evidence has shown that conventional normalization with a single gene might lead to inaccurate qRT-PCR results [35,41] and that a combination of multiple reference genes provides greater precision. In the present study, *EsDREB2* expression in plants exposed to drought stress was compared using *EsEF* or *Esα-TUB* alone or the combination of *EsEF* and *Esα-TUB*. When *EsEF* was used alone or *EsEF* and *Esα-TUB* were used together for normalization, *EsDREB2* gene expression showed a similar pattern. *EsDREB2* expression increased in response to drought stress after 2 h, reached a maximum by 6 h and then declined at 12 h (Figure. 5). This expression profile for *EsDREB2* is similar to that in other plants including *Populus euphratica* [51]. When *Esα-TUB* was used alone for normalization, *EsDREB2* transcript levels appeared essentially unchanged from 0 h (control) to 12 h and then declined after 48 h. This result further confirmed the importance of carefully selecting more than one stable and suitable reference gene for accurate qRT-PCR normalization.

## 4. Experimental Section

### 4.1. Plant Materials and Treatments

*Eremosparton songoricum* (Litv) Vass. seeds were collected from the Gurbantunggut Desert, within the Xinjiang Uygur autonomous region of the P. R. China (88°24′67′′E, 45°58′11′′N, 667m asl), and were soaked in 98% (*v/v*) sulfuric acid for 10-15 min to break the physical dormancy of seeds followed by washing with ddH_2_O. Seeds were placed on moist filter paper in petri-plates, and seedlings were grown at 25 °C with cycles of 12 h light and 12 h darkness (100 μmol m^−2^·s^−1^) and 60% relative humidity prior to stress treatments.

We exposed *E. songoricum* to nine different stress conditions to evaluate the stability of the tested reference genes. The stress treatments included common stresses experienced by this species in its natural environment (e.g., salinity, drought, grazing) and more common stresses that alter gene expression in plants in general (e.g., free radicals or oxidative potential, heavy metal exposure, and ABA). For NaCl, PEG, ABA, oxidation and metal treatments, 2-week-old seedlings were placed on filter paper saturated with 8 mL of the following solutions: 250 mM NaCl, 20% (*w/v*) polyethylene glycol (PEG6000), 100 μM ABA, 50 mM H_2_O_2_, 0.5 mM CuSO_4_ and 0.5 mM ZnSO_4_, respectively. For cold and heat treatments, 2-week-old seedlings were incubated at 4 °C and 40 °C, respectively. For UV radiation treatment, 2-week-old seedlings were exposed to 0.5 w/m^2^ UV-B irradiation. For mechanical wounding, leaves were cut six times per leaf with a razor blade [8]. Two-week-old seedlings grown under identical conditions and handled in a similar manner served as the control (e.g., control plants were placed onto water-saturated filter paper). For all stress treatments described above, tissue samples (e.g., leaves, stems and roots) were harvested after 4 hours. For comparison with laboratory-grown *E. songoricum* materials, field-grown adult roots, assimilating branches and flowers were collected from the same location as the seed source described above (the Gurbantunggut Desert). Because seed germination is one of the most important stages for stress resistance [52], additional seeds were placed onto moist filter paper placed in petri-plates (as described above), and whole seeds were collected on days 1, 3 and 7 after germination. After harvest, all samples were flash frozen in liquid nitrogen and stored at −80 °C prior to RNA extraction.

### 4.2. RNA Extraction and cDNA Synthesis

Total RNA extraction was performed using RNAiso™ plus reagent (Takara, Japan) following the manufacturer’s instructions with little modification. Genomic DNA contamination was eliminated using RNase-free DNaseI (Takara, Japan). RNA concentration, purity, and integrity were determined using a NanoDrop ND-1000 spectrophotometer (Thermo Fisher Scientific, USA) and visually assessed via gel electrophoresis (1.2% agarose). Only RNA samples with a 260/280 ratio between 1.9 and 2.1 and 260/230 ratio higher than 2.0 were used for subsequent analyses. First strand cDNA was synthesized from 1 μg total RNA, l μL oligo-dT, 1 μL random hexamers and 4 μL 5× Primerscript Buffer (Takara, Japan). The RT-PCR reaction was carried out at 37 °C for 30 min on a C1000™ Thermal cycler (Bio-Rad, USA) in a final volume of 20 μL, and inactivation of the enzyme was achieved at 85 °C for 5 min. Prior to qRT-PCR, cDNA samples were tested at templates by RT-PCR for the *ACT* gene (data not shown). Finally, all cDNA was stored at −20 °C.

### 4.3. Cloning the Partial Sequences of the Candidate Reference Genes

We selected the following seven classic reference genes spanning a range of biological functions as reference gene candidates in *E. songoricum*: 18S ribosomal RNA (*18S*), elongation factor 1 alpha (*EF*), cytoskeletal structural protein (*ACT*), ubiquitin protein (*UBQ*), glyceraldehyde-3-phosphate dehydrogenase (*GAPDH*), and the cytoskeletal structural proteins α-tubulin (*α-TUB*) and β-tubulin (*β-TUB*). The primers for *ACT*, *18S*, *β-TUB* and the gene of interest *EsDREB2* were designed with Primer Premier 5.0 with the mRNA sequences of *ACT* (EU529707.1), *18S* (AF293760.1), *β-TUB* (U12286.1) and *EsDREB2* of *E. songoricum* (HQ687367). The primers for *EF*, *UBQ*, *GAPDH* and *a-TUB* were based on the literatures [43,53–55]. The information on the primers for the reference genes is provided in supplemental material (Table S1).

PCR was performed using TaKaRa Taq™ (TaKaRa, Japan), and the reaction conditions were as follows: 30 cycles with denaturation at 94 °C for 30 s, annealing at 55 °C for 30 s, and extension at 72 °C for 45 s. An initial denaturation step of 3 min at 95 °C and a final elongation step at 72 °C for 7 min were performed. The final amplification products were checked on a 1% (*w/v*) agarose gel in 1× TAE buffer. Amplicons were purified using the EZ Spin Column DNA Gel Extraction Kit (BIO BASIC INC, Canada) and cloned into the pMD™19-T vector according to the manufacturer’s instructions. Positive colonies containing recombinant plasmids were then sent to the Beijing Genomics Institute (Beijing, China) for DNA sequencing. The resulting DNA and deduced polypeptide sequences were used to query the appropriate databases using the BLAST algorithm [56,57] of the National Center for Biotechnology Information (NCBI).

### 4.4. QRT-PCR Primer Design and Testing

Based on the gene sequences obtained from homology cloning, eight gene-specific primer sets were designed for qRT-PCR (Table 1). Specifically, primers were designed with Primer Premier 5.0 using the following as criterion: amplicon length from 100 to 300 bp and a *T*m of 58 ± 1 °C. Amplification efficiency (*E*) was evaluated using a standard curve generated by qRT-PCR using a ten-fold dilution series over at least four dilution points that were measured in triplicate. Primer specificity was confirmed using melting-curve analysis after qRT-PCR and gel electrophoresis analysis of the amplicon. For a more detailed explanation of all qRT-PCR experiments, see the MIQE guidelines proposed by Bustin [58].

### 4.5. Quantitative Real-Time PCR

All cDNA samples were diluted 5-fold with RNase-free water before being used as templates in quantity analysis. Real-time PCR reactions using SYBR Premix Ex Taq™ (Takara, Japan) were performed using the CFX96 Real-Time PCR Detection System (Bio-Rad, USA) with 96-well plates. The reaction mixture consisted of 2 μL 1:5 diluted cDNA samples, 0.4 μL each of the forward and reverse primers (10 μM), 10 μL real-time master mix and 7.2 μL PCR-grade water in a final volume of 20 μL. Two biological replicates for all of the samples and three technical replicates of each biological replicate with a no-template control (NTC) were also used. The RT-PCR protocol was as follows: 30 s initial denaturation at 95 °C, 40 cycles of 94 °C for 5 s and 60 °C for 30 s. To verify the specificity of each primer, a melting-curve analysis was included (65 °C to 95 °C with fluorescence measured every 0.5 °C). Data were analyzed using Bio-Rad CFX Manager software (version 1.6) (Bio-Rad, USA).

### 4.6. Statistical Analysis of Gene Expression Stability

GeNorm v. 3.5 (a publicly available software tool) was used to rank and analyze the stability of expression of the reference genes in each of the samples [15]. According to the geNorm manual, the *Cq* values were imported into Microsoft Excel and transformed into relative quantities using the formula *Q* = (1 + *E*) ^(min^ *^Cq^* ^− sample^ *^Cq^*^)^ and then imported into geNorm (version 3.5). The expression stability value *M* and pairwise variation value *V* for each reference gene with all other genes were automatically analyzed and ranked according to their expression stability. Then, the optimal number of reference genes for normalization was determined.

### 4.7. Normalization of a Gene of Interest, EsDREB2

*EsDREB2* was cloned from our lab (GenBank No HQ687367) (data not shown). To assess the validity of the procedure for the selection of reference genes detailed above, we evaluated the relative expression level of *EsDREB2*. The expression of the *EsDREB2* gene was evaluated across five samples: 0 h (control), 2 h, 6 h, 12 h, and 48 h after drought treatment. The expression of *EsDREB2* was analyzed by Bio-Rad CFX Manager software (version 1.6) (Bio-Rad, USA) and is shown as the mean ± SE of three replicates for each sample.

## 5. Conclusions

In summary, our data show that expression stability varied between reference genes in different experimental setups and that no single reference gene is suitable for accurate normalization of qRT-PCR.

1. For all the tested samples, *ACT* and *β-TUB1* were the most stable reference genes, and seven genes were needed for accurate qRT-PCR normalization for the experimental design of all samples (V_7/8_ = 0.163).
2. For the different tissues and developmental stage subgroups, *EsACT*, *Esβ-TUB2* were the most suitable reference genes, and the addition of the *EsUBQ* gene was necessary for a reliable normalization result (V_3/4_ = 0.168)
3. For untreated plants or stress-treated samples, including NaCl, PEG, ABA, cold, heat, UV, oxidation, injury and metal treatments, *EsEF* and *Esα-TUB* were the most stable genes, and the combination of the two was sufficient for normalization (V_2/3_ = 0.147)
4. For seedling tissues grown under controlled conditions, *EsEF* and *Esβ-TUB2* were the most stable reference genes and were sufficient for normalization (V_2/3_ = 0.087). For adult plant tissues collected from the field, *EsGAPDH* and *EsUBQ* were the two most stable genes and are sufficient for normalization (V_2/3_ = 0.113)
5. Comparing tissue of seedlings grown under controlled conditions and adult plants collected from the field, *EsACT* and *Esβ-TUB2* were the most stable genes, but the addition of another two or more stable genes is recommended for better normalization (V_4/5_ = 0.186)
6. For the different stages of germination, *EsEF* and *EsACT* were the most stable genes, and these two genes were sufficient for normalization (V_2/3_ = 0.05).
7. The *Es18S* gene was found to be unsuitable as a reference gene in our analysis.

To our knowledge, this is the first report for the isolation and evaluation of qRT-PCR reference genes from *E. songoricum*. This study will provide the foundation for large-scale selection of reference genes in this species, and facilitate future work on gene expression studies in *E. songoricum* and other Leguminosae shrubby plants.

## Figures and Tables

**Table 1:** Primer sequences and amplicon characteristics of eight reference genes and one gene of interest for quantitative real-time reverse transcriptase polymerase chain reaction (qRT-PCR) analysis.

Gene	Accession number	Primer sequence (5′→3′, forward/reverse)	Length (bp)	Amplificatin efficiency (%)	*R*^2^	Amplicon *T*m (°C)
*GAPDH*	JN866814	AATGTCTTTCCGTGTCC / TCCTCTTCAATGTAACCC	150	93.5	0.993	81
*UBQ*	JN866815	TGGTCGCACCTTAGCCG / TCCCTCCTTATCTTGAATCTTGG	178	91.5	0.999	82
*ACT*	JN866816	AGGAACCACCGATCCAGACA / GGTGCCCTGAGGTCCTGTT	275	92.9	0.995	83
*EF*	JN866817	CGGACATCGTGACTTTATC / TGTGGTGGCATCCATCTT	199	94.9	0.992	82.5
*β-TUB1*	JN866818	ATTCCTTTCCCTCGTTTG / AATGTGGGATGCCAAGAA	122	99.5	0.999	83
*β-TUB2*	JN866819	TTACCTCACCGCCTCAG / AAGCCATCTTCAAACCT	167	93	0.999	80.5
*α-TUB*	JN866820	TAGCAGCGTCTTCCTTT / ATGGTTTGATGCCGAGT	204	98.9	0.997	84
*18S*	JN866821	GGAGAGGGAGCCTGAGA / CACCAGACTTGCCCTCCAA	188	98.4	1	83.5
*EsDREB*	HQ687367	GCTGCTCTTGCTTATGAT / TCTACCCCCGAGTTGTTT	185	94.9	0.999	84.5

**Table 2:** The most and least stable reference genes for each of the specific experimental conditions.

Experiment sets	The three most stable genes	Optimal combination	The least stable gene
total	*ACT*, *β-TUB1*, *β-TUB2*	V_7/8_ = 0.163	*18S*
tissues and developmental stage	*ACT*, *β-TUB2*, *UBQ*	*ACT + β-TUB2 + UBQ* (V_3/4_ = 0.168)	*18S*
stresses	*EF*, *α-TUB, UBQ*	*EF + α-TUB* (V_2/3_ = 0.147)	*GAPDH*
tissues	*ACT*, *β-TUB2*, *UBQ*	(V_4/5_ = 0.186)	*18S*
tissues (seedling)	*EF*, *β-TUB2*, *ACT*	*EF + β-TUB2* (V_2/3_ = 0.087)	*18S*
tissues (adult)	*GAPDH*, *UBQ*, *β-TUB2*	*GAPDH* + *UBQ* (V_2/3_ = 0.113)	*18S*
germination stage	*EF*, *ACT*, *α-TUB*	*EF + ACT* (V_2/3_ = 0.05)	*GAPDH*
